# A Survey of Oncologists’ Perceptions and Opinions Regarding Brain Imaging in Metastatic Renal Cell Carcinoma Within the UK

**DOI:** 10.7759/cureus.103513

**Published:** 2026-02-12

**Authors:** Ben T Crosby, Emmanuel Eloebhose, Muhammad Asad Saeed, Maneha Sethi, Steffanie Winsor, Alice Spurr, Jose Tapia, Will Ince, Ricky Frazer, John McGrane

**Affiliations:** 1 General Medicine, Morriston Hospital, Swansea, GBR; 2 General Medicine, Velindre Cancer Centre, Cardiff, GBR; 3 Oncology, Velindre Cancer Centre, Cardiff, GBR; 4 Internal Medicine, University Hospital Wales, Cardiff, GBR; 5 Internal Medicine, Morriston Hospital, Swansea, GBR; 6 Oncology, Addenbrookes Hospital, Cambridge, GBR; 7 Immuno-Oncology, Velindre Cancer Centre, Cardiff, GBR; 8 Oncology, Royal Cornwall Hospital, Truro, GBR

**Keywords:** brain metastases, imaging, renal cell carcinoma, surveillance, survey, survey research

## Abstract

Brain metastases are an important cause of morbidity and mortality in patients with advanced renal cell carcinoma (aRCC). Despite emerging evidence suggesting a clinically meaningful prevalence of asymptomatic brain metastases, there remains no consensus regarding routine central nervous system (CNS) imaging in this population. This questionnaire study aimed to explore current opinions and management practices relating to brain imaging for aRCC across the UK.

A nationwide, web-based survey was conducted using Google Forms between June and October 2025, targeting UK oncology consultants with a subspecialist interest in renal cell carcinoma (RCC). The questionnaire assessed demographics, guideline use, current CNS imaging practices, and willingness to change practice. Fifty consultants were invited to participate, and 25 responses were received, representing a 50% response rate from multiple centres across the UK.

Most respondents (68%) reported not following any specific guideline for CNS imaging in patients with aRCC. Considerable variation in clinical practice was observed. Nearly half of respondents (48%) reported performing CNS imaging only in the presence of neurological symptoms, while 28% performed routine surveillance imaging in asymptomatic patients. Smaller proportions reported imaging patients with high-risk features at diagnosis (12%), incorporating CNS imaging into baseline staging for metastatic disease (4%), or performing imaging only at disease progression or change in systemic therapy (8%). Imaging modality and surveillance frequency also varied widely. Notably, 92% of respondents stated they would consider adopting routine CNS imaging for all patients with aRCC if evidence demonstrated improved clinical outcomes.

This survey demonstrates substantial variability in CNS imaging practices for patients with aRCC among UK oncology consultants, reflecting the absence of standardised guidance. The strong willingness to change practice highlights the need for prospective studies to clarify the clinical value, optimal timing, and cost-effectiveness of routine brain imaging. Robust evidence is required to inform evidence-based guidelines, reduce practice variation, and support consistent, patient-centred care for patients with aRCC.

## Introduction

Renal cell carcinoma (RCC) accounts for approximately 4% to 5% of adult malignancies and represents the majority of kidney cancers [1]. Approximately 30% of patients present with advanced renal cell carcinoma (aRCC), which is associated with a historically poor five-year survival rate of approximately 8% [2]. While cytokines and tyrosine kinase inhibitors (TKIs) once dominated systemic therapy, immune checkpoint inhibitors have reshaped the therapeutic landscape and dramatically improved survival outcomes [3].

Brain metastases represent a critical site of disease progression in patients with aRCC, and are associated with reduced overall survival [4]. The reported incidence of brain metastases ranges from 2% to 16% [4-6]. This variation can be attributed, at least in part, to the fact that patients are typically diagnosed only when new neurological symptoms prompt further investigation. As a result, historical incidence estimates may under-represent asymptomatic disease, limiting their clinical utility in the absence of established screening strategies. A study by Kotecha et al. in 2021 [6] suggested that baseline brain imaging should be considered in patients with aRCC, reporting that 4.3% of 1,689 patients presented with asymptomatic brain metastases at diagnosis. Localised brain-directed therapy was administered to 93% of these patients, predominantly radiotherapy [6].

Recent advances in both local and systemic therapies for extracranial disease have extended survival in patients with aRCC, thereby increasing the likelihood of developing brain metastases over the course of the disease [7]. In part due to this emerging evidence, along with improved detection and treatment of small intracranial lesions using stereotactic radiosurgery (SRS) [8] and personalised systemic therapies, clinical practice guidelines from the European Society for Medical Oncology (ESMO) and the National Comprehensive Cancer Network (NCCN) recommend that baseline neuroimaging (CT or MRI) be considered in all cases of metastatic disease before the initiation of systemic therapy [9,10]. However, the frequency of subsequent imaging is left to a physician's discretion and guided by clinical symptoms, resulting in limited standardisation of central nervous system imaging protocols for patients with RCC. The devised questionnaire aims to provide insight into current opinions and management practices regarding brain imaging in patients with aRCC across the UK.

## Materials and methods

Survey design

This study was conducted as a questionnaire-based cross-sectional study aimed at oncology consultants within the UK. The survey was developed using Google Forms (Google LLC, Mountain View, CA, USA) between June and October 2025 and targeted UK oncology consultants. The survey was developed and piloted at the 2025 UK Oncology Forum. Based on feedback from this pilot, the original survey was modified to ensure completeness and ease of use. Although the instrument was piloted to optimise clarity and feasibility, it did not undergo formal validation (content‑validity assessment, construct validation, or reliability testing). Thus, it constitutes a limitation inherent to exploratory questionnaire studies and means that differential interpretation of items by respondents cannot be excluded.

The final survey comprised 17 questions: 15 quantitative questions (including yes/no questions (n = 2) and multiple-choice questions (n = 13)) and two qualitative free-text questions. Questions were divided into four major topics: demographics, current guidelines, current practice, and willingness to change. In addition to these four topics, a free-text question was included to solicit further comments and ensure completeness. To minimise missing data, the survey was designed so that it could not be submitted unless all questions were answered. An overview of the survey questionnaire is included in Appendix A.

Sampling and study dissemination

This study employed a census sampling approach with accessible UK oncology consultants with a subspecialist interest in RCC. Rather than selecting a subset of participants, the intention was to invite all eligible consultants who could be reached through established professional channels. Eligible participants were identified via existing UK oncology email distribution lists and through attendance at the UK Renal Oncology Collaborative (UK ROC) national meeting in London.

The target sample size of 50 consultants was determined pragmatically, based on the estimated number of eligible consultants accessible through these channels, rather than through a formal sample size or power calculation. This approach was considered appropriate given the specialist nature of the population and the exploratory aim of the study. The primary aim of the sampling strategy was to maximise participation from oncology consultants across the UK to capture a broad geographical spread and to best reflect consensus opinion within this specialist clinical group.

Data collection and analysis

The questionnaire was clinician-directed and designed to capture centre- and clinician-level imaging practice. It did not collect patient-level tumour characteristics (e.g., histological grade, primary tumour size or burden), detailed institutional staging protocols, or individual genetic/syndromic data. The collated data were analysed at the Velindre Cancer Centre (Cardiff, GB-WLS) using SPSS Statistics version 30 (IBM Corp., Armonk, NY, USA).

Ethics statement

The present study was conducted through an anonymous, voluntary survey targeting medical professionals to elicit their professional opinions and practices. All participants provided informed consent and were adequately informed that the survey results would be submitted for publication. No financial incentives or other payments were offered for survey completion. Importantly, the study did not involve any patient participation, nor did it contain identifiable personal data. In accordance with institutional policy and applicable regulations, this research was deemed exempt from formal application to the Integrated Research Application System (IRAS) or Institutional Review Board (IRB) review. Consequently, following the local institutional policies at Velindre Cancer Centre, formal approval was not required. The study was carried out in adherence to local guidelines and in compliance with data protection regulations.

## Results

Demographics

Twenty-five consultants and specialty doctors responded to the survey, representing a response rate of approximately 50% (25/50). Respondents were geographically distributed across the UK, representing 21 different trusts within the NHS. Participants reported varying numbers of consultants within their local trusts. Respondents also varied in their level of clinical experience and in the number of new RCC patients treated per month. The demographic characteristics of the study cohort are summarised in Table 1.

**Table 1:** Demographic characteristics of the participating group RCC: Renal cell carcinoma

Demographic variable	Item	Frequency
Job role	Consultant	24 (96%)
	Specialty Doctor	1 (4%)
NHS trust	Cambridge University Hospitals Teaching Trust	1 (4%)
	Hull University Teaching Hospitals NHS Trust	1 (4%)
	Cambridge University Hospitals Trust	1 (4%)
	Belfast Health and Social Care Trust	1 (4%)
	Beatson West of Scotland Cancer Centre	1 (4%)
	Leeds Teaching Hospitals NHS Trust	1 (4%)
	East and North Hertfordshire NHS Trust	1 (4%)
	Newcastle Hospitals NHS Foundation Trust	1 (4%)
	NHS Lothian	1 (4%)
	Royal Cornwall Hospitals NHS Trust	2 (8%)
	Royal Free London NHS Foundation Trust	1 (4%)
	Lancashire Teaching Hospitals Trust	1 (4%)
	Royal Devon University Healthcare NHS Foundation Trust	1 (4%)
	Shrewsbury and Telford Hospital NHS Trust	1 (4%)
	University Hospital Southampton NHS Foundation Trust	1 (4%)
	Torbay & South Devon NHS Foundation Trust	1 (4%)
	University Hospital North Midlands NHS Trust	2 (8%)
	United Lincolnshire Hospitals NHS Trust	1 (4%)
	University Hospitals Birmingham NHS Foundation Trust	1 (4%)
	Velindre Cancer Centre NHS Trust	3 (12%)
	Sheffield Teaching Hospitals NHS Foundation Trust	1 (4%)
Number of consultants working in the current organisation	1	1 (4%)
	2	4 (16%)
	3	10 (40%)
	4	5 (20%)
	5	2 (8%)
	>5	3 (12%)
Length of time in current role (years)	<5 years	10 (40%)
	5-10 years	5 (20%)
	11-15 years	6 (24%)
	16-20 years	2 (8%)
	21-25 years	2 (8%)
The average number of new RCC patients treated by the participant per month	<4	5 (20%)
	4-6	7 (28%)
	7-9	6 (24%)
	>9	7 (28%)

Current guidelines

**Figure 1:**
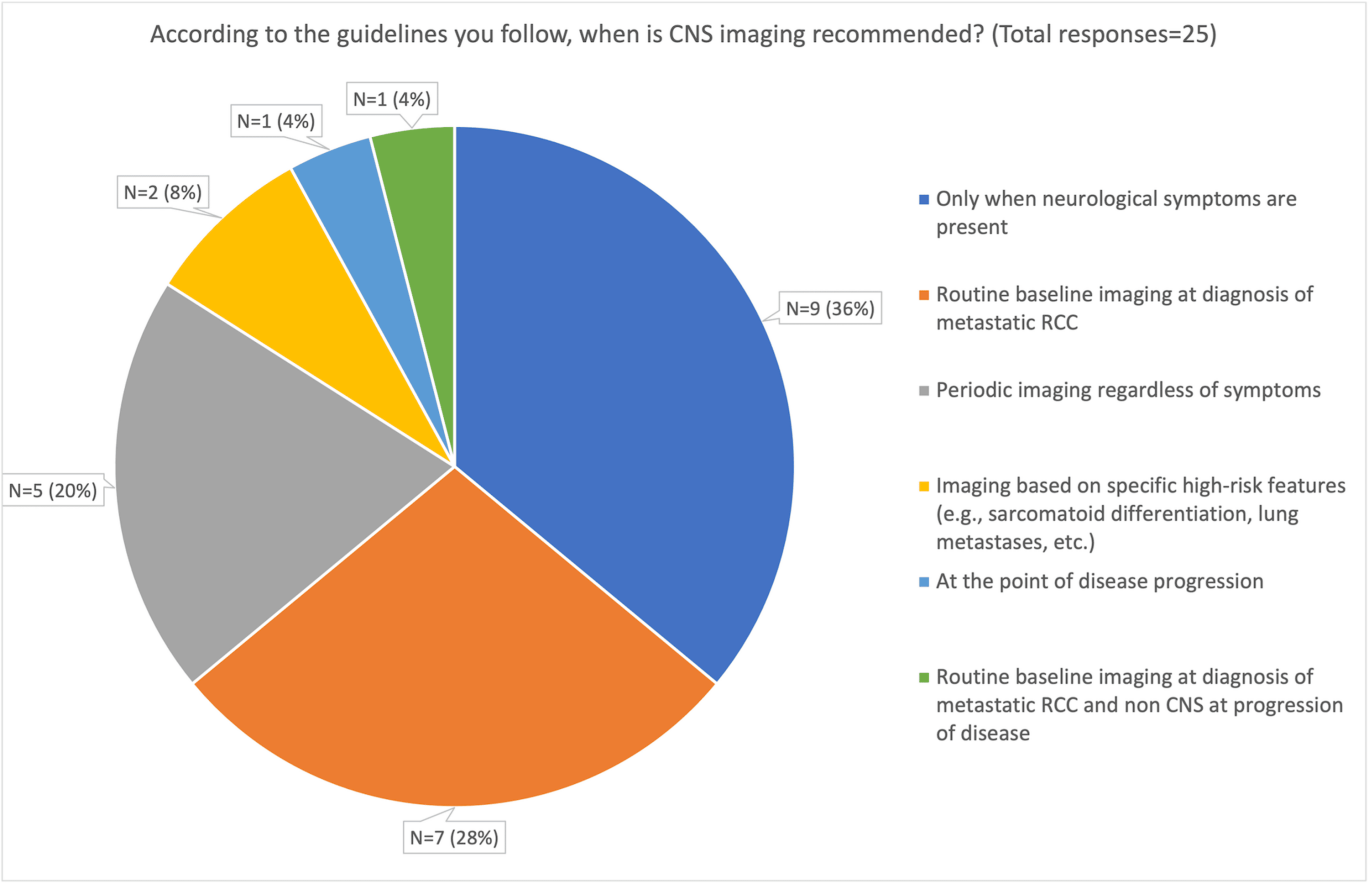
Pie diagram of questionnaire responses per the reported recommended frequency of CNS imaging according to clinicians’ usual guidelines RCC: Renal cell carcinoma, CNS: Central nervous system

Our results demonstrate that the majority of consultants (17/25, 68.0%) do not follow any specific guideline, while a further 7/25 (28.0%) follow institutional or local guidelines. Only one consultant (4.0%) reported adherence to the ESMO guidelines [9]. Respondents were also asked about imaging frequency based on the guidelines they followed. Considerable variation in imaging practices was observed, with the most common approaches being imaging only in the presence of neurological symptoms (36%, n = 9) or routine baseline imaging at the time of metastatic RCC (aRCC) diagnosis (28%, n = 7). These findings are summarised in Figure 1.

Current practice

**Figure 2:**
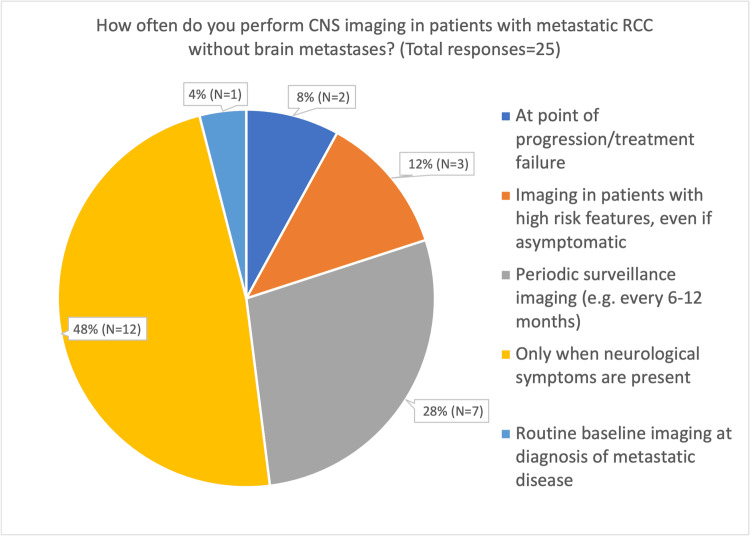
Pie chart of responses by the typical reported frequency of brain imaging

Our results show that, within their own practice, respondents reported performing CNS imaging for patients with metastatic RCC without known brain metastases at varying frequencies. A significant number of consultants reported imaging patients only when neurological symptoms were present (12/25, 48%), while 7/25 (28.0%) reported imaging at regular intervals. Three consultants (3/25, 12.0%) reported imaging patients with high-risk features at the point of diagnosis, and one consultant (1/25, 4.0%) reported utilising CNS imaging as part of baseline staging for metastatic disease. Two consultants (2/25, 8.0%) reported imaging the brain only at the point of disease progression or when changing lines of treatment (Figure 2).

**Figure 3:**
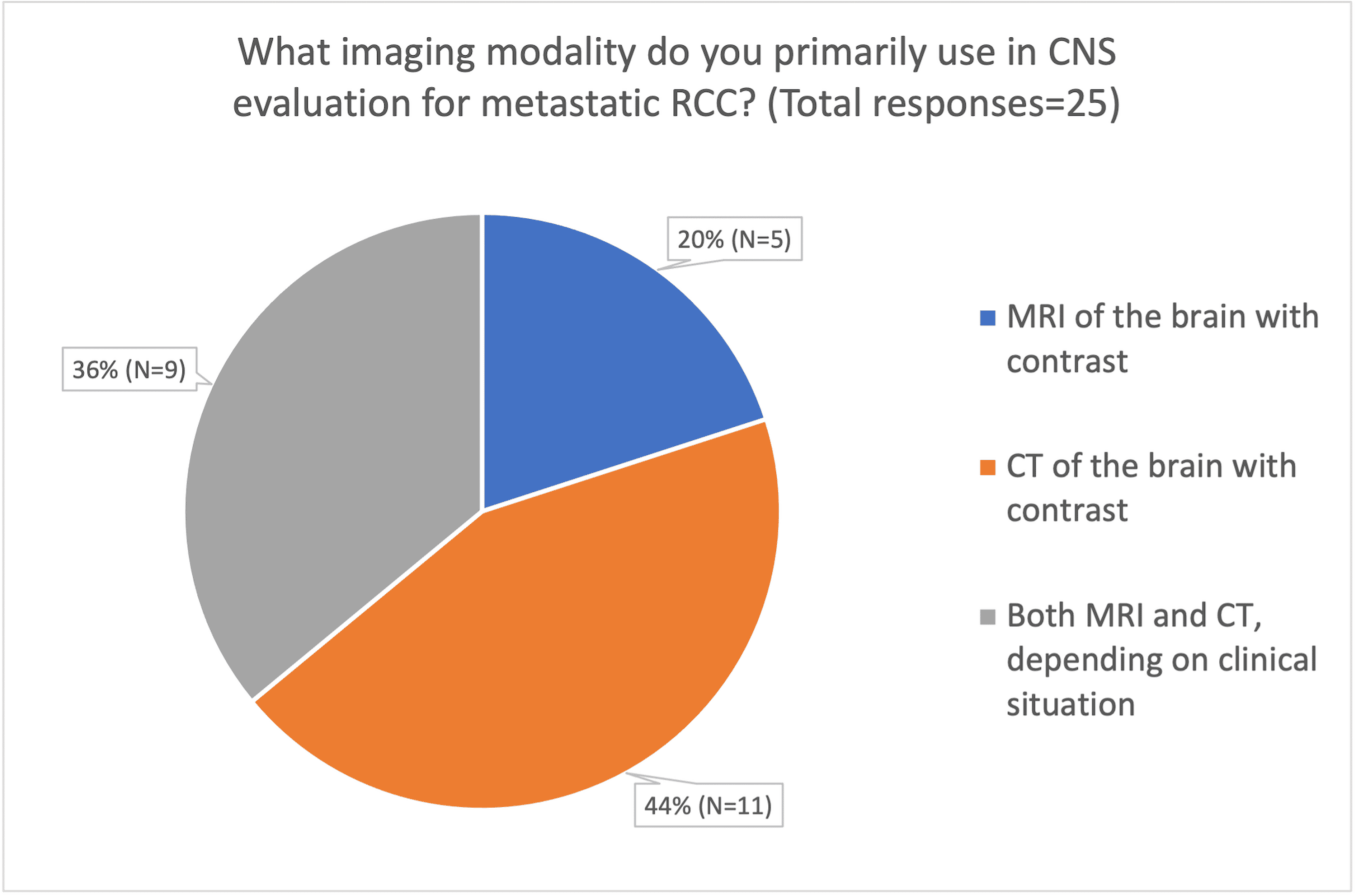
Pie chart detailing the primary imaging modality of choice for periodic CNS surveillance CNS: Central nervous system

**Figure 4:**
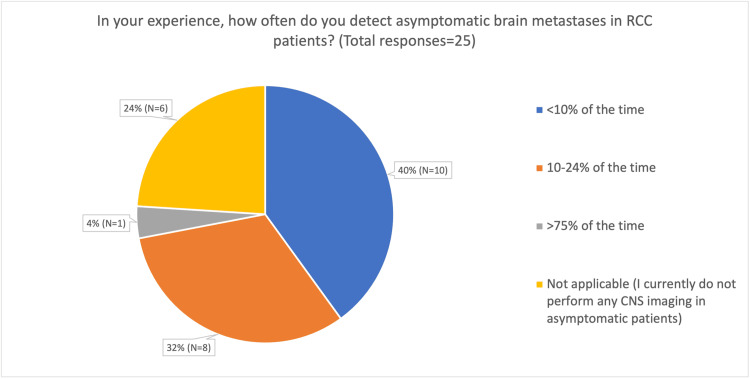
Pie chart showing the estimated detection rate of asymptomatic brain metastases in respondents’ own practice RCC: Renal cell carcinoma, CNS: Central nervous system

In evaluating brain metastases, respondents reported using both contrast-enhanced CT and MRI (Figure 3). Specifically, 36% (n = 9) chose to use both modalities as needed, 20% (n = 5) preferred MRI, and 44% (n = 11) initially utilised CT scans of the head. All respondents who reported using MRI with contrast as the initial imaging modality did so only when neurological symptoms were present. When utilising routine periodic surveillance, respondents reported varying experiences regarding the rate of detection of asymptomatic brain metastases in RCC patients, ranging from less than 10% of cases to more than 75% of cases (Figure 4).

Willingness to change

**Figure 5:**
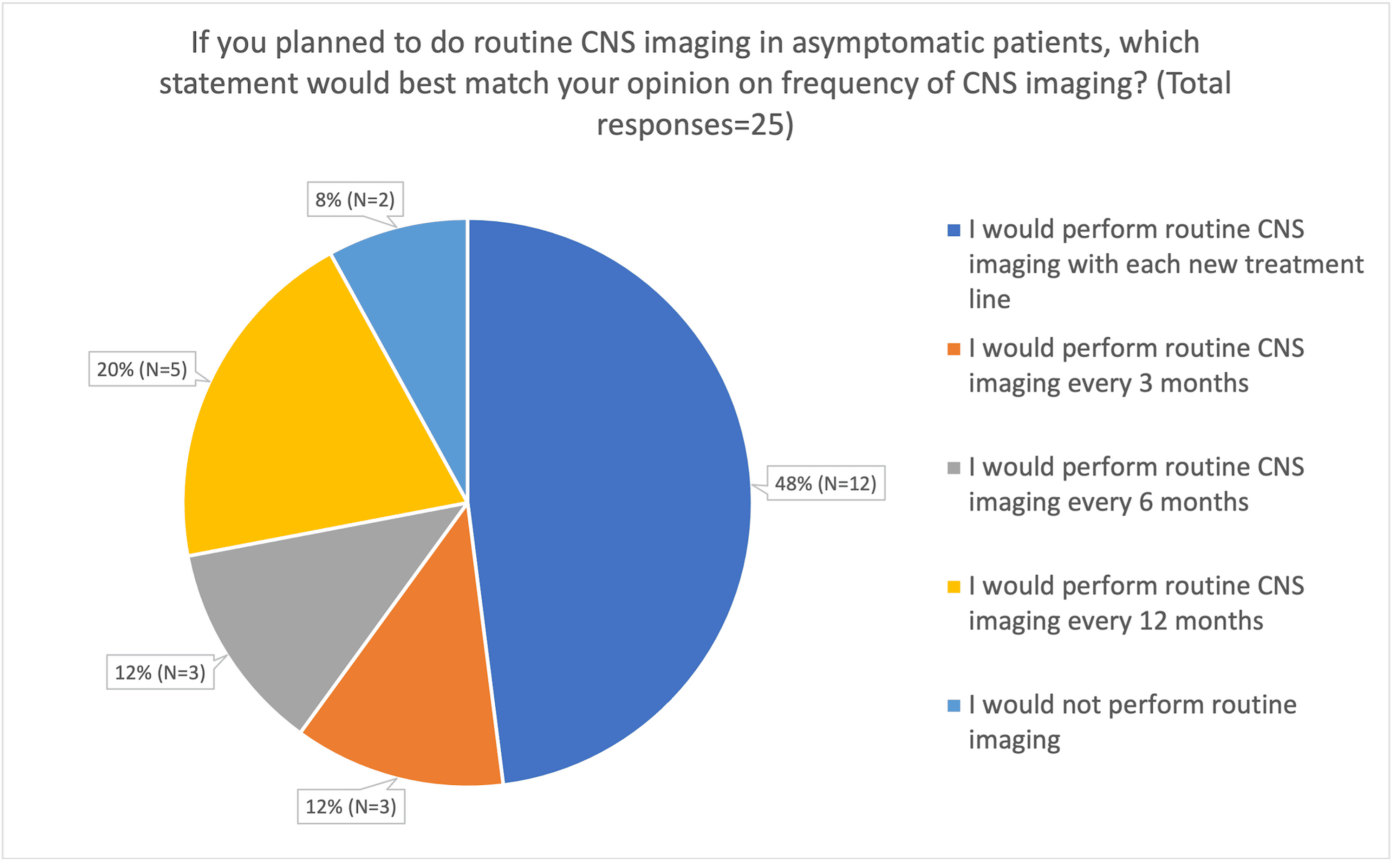
Pie chart showing the theoretical imaging frequency for routine CNS imaging in asymptomatic patients with aRCC CNS: Central nervous system, aRCC: Advanced renal cell carcinoma

The majority of respondents stated that they would consider adopting routine CNS imaging for all those with aRCC if evidence suggested it improved outcomes (yes: 23/25 (92%); maybe (depending on strength of evidence): 2/25 (8.0%)). When questioning respondents specifically regarding opinions on the frequency of asymptomatic imaging and if they were going to perform periodic surveillance of CNS metastases, a wide variety of answers were reported. Of the total participants, 12/25 (48.0%) reported that they would perform imaging with each new line of treatment; 5/25 (20.0%) stated they would perform imaging every 12 months; 3/25 (12.0%) every six months; 3/25 (12.0%) stated they would perform imaging every three months; and 2/25 (8.0%) stated that they would not perform routine imaging (Figure 5).

Respondents reported multiple factors that would influence the decision to change CNS imaging practices and highlighted numerous barriers/reasons against doing routine brain imaging in asymptomatic patients. These factors are displayed in Table 2. 

**Table 2:** Factors that would influence the decision to change CNS imaging practices CNS: Central nervous system

Factors that would influence your decision to change CNS imaging practices	Number of responses (Total n = 25)
Updated national international guideline recommendations	20 (80%)
Strong clinical trial evidence supporting routine imaging	15 (60%)
Evidence showing the cost-effectiveness of routine imaging	11 (44%)
Improved access to resources (e.g., MRI availability)	8 (32%)
Feedback from multidisciplinary teams	5 (20%)
Patient preference for more comprehensive imaging	5 (20%)
Reasons for not doing increased frequency CNS imaging	Number of responses (total n=25)
Logistical challenges	10 (40%)
Cost and resource allocation	8 (32%)
No reason	8 (32%)
Lack of clear benefit to survival or quality of life	7 (28%)
Increased patient anxiety	6 (24%)
Overdiagnosis leading to unnecessary investigations	2 (8%)
Concern regarding driving rules/Driver and Vehicle Licensing Agency (DVLA) restriction	1 (4%)

Responses from outside the UK

Despite the survey being aimed at UK participants at national conferences, an additional seven responses were received from consultants based outside the UK. Although these responses were excluded from the main analysis, they indicated that the lack of standardisation in CNS imaging is not unique to the UK. Responses came from Croatia (n = 1/7, 14.3%), Italy (n = 1/7, 14.3%), France (n = 1/7, 14.3%), Iraq (n = 1/7, 14.3%), Germany (n = 1/7, 14.3%), Turkey (n = 1/7, 14.3%), and Nigeria (n = 1/7, 14.3%), demonstrating varied practice. Three consultants (3/7, 42.9%) reported performing CNS imaging in patients with metastatic RCC without known brain metastases only when neurological symptoms were present, while three (3/7, 42.9%) reported periodic surveillance imaging (e.g., every six to 12 months). One consultant (1/7, 14.3%) reported performing routine baseline CNS imaging at the diagnosis of metastatic disease.

## Discussion

We surveyed 25 consultants from multiple centres across the UK to evaluate current practices in CNS imaging for aRCC. Our findings demonstrate substantial heterogeneity in clinical practice. Notably, 68% (17/25) of respondents reported not following any specific guideline for brain imaging in aRCC, highlighting marked variability despite existing recommendations from the ESMO [9] and the NCCN [10]. Both guidelines recommend CNS imaging primarily in the presence of neurological symptoms or specific clinical suspicion and do not endorse routine serial CNS imaging or define a standard imaging frequency for asymptomatic patients.

This variability likely reflects the historically discretionary nature of guideline recommendations for asymptomatic CNS imaging in RCC, driven in part by the perceived low incidence of occult brain metastases [11,12]. While current guidelines continue to support selective imaging, contemporary multi-institutional prospective data suggest that approximately 4.3% of asymptomatic patients with aRCC harbour occult brain metastases, including those with favourable International Metastatic RCC Database Consortium (IMDC) risk profiles [6,13]. These emerging data challenge the assumption that CNS involvement is uncommon in the absence of neurological symptoms and raise the possibility that broader imaging strategies may be warranted.

Early detection of small or limited brain metastases has important therapeutic implications, as such lesions are increasingly amenable to effective local and systemic treatments [13-15]. Stereotactic radiosurgery (SRS) achieves high local control rates in RCC brain metastases, particularly for small-volume disease, with acceptable toxicity and preservation of neurological function [16]. In addition, contemporary series indicate that the combination of SRS with targeted systemic therapies is associated with improved survival and durable intracranial disease control [17]. Recent reviews further highlight that, in the era of modern systemic agents and precision radiotherapy, proactive management of limited CNS disease may help preserve neurological function and quality of life while extending overall survival [18].

The survey also revealed considerable variation in the reported frequency of CNS imaging. Some consultants (5/25, 20%) reported performing regular CNS imaging in neurologically asymptomatic patients irrespective of tumour biology, whereas others imaged only at systemic disease progression (1/25, 4%), only in the presence of high-risk features (2/25, 8%), or exclusively when neurological symptoms developed (9/25, 36%). These findings suggest that clinical judgement and patient-specific factors play a significant role in decision-making in the absence of definitive guidance.

With respect to imaging modality, 44% of respondents (11/25) reported using CT for CNS assessment, 36% (9/25) primarily used contrast-enhanced MRI, and 20% (5/25) used both modalities depending on clinical circumstances. While CT is widely available and commonly incorporated into routine systemic staging, contrast-enhanced MRI is generally regarded as the superior modality for detecting brain metastases due to its higher sensitivity and specificity, particularly for small lesions and those located in the posterior fossa [19]. Differences in access, service demand, and local imaging protocols likely contribute to this variability. Notably, consultants who primarily used MRI tended to reserve it for symptomatic patients, possibly reflecting either a strategy to limit MRI to cases requiring detailed neuroanatomical assessment or constraints related to MRI availability.

Our data also demonstrate a strong willingness among respondents to modify practice in the presence of robust supporting evidence. The majority indicated that updated national or international guideline recommendations would be the most influential factor driving a change in practice (80%, n = 20), underscoring the central role of formal guidance in clinical decision-making. This finding highlights the potential for future high-quality studies to meaningfully influence practice patterns. Factors most likely to facilitate change included updated guidelines, emerging evidence of clinical benefit, and improved access to imaging resources. Although practical considerations were comparatively less influential overall, logistical challenges were the most frequently cited barrier to increased imaging (40%, n = 10). While only one-third of respondents identified improved access to resources, such as MRI availability, as a driver of change (32%, n = 8), these findings suggest that addressing logistical and resource constraints may still play an important enabling role.

In contrast, 28% of respondents (7/25) expressed concern regarding the lack of evidence demonstrating a clear survival or quality-of-life benefit associated with increased CNS imaging. Psychosocial considerations, including increased patient anxiety, overdiagnosis, unnecessary investigations or interventions, and additional healthcare costs, were also highlighted. Such concerns are increasingly recognised in oncology, where clinicians must balance the potential advantages of early detection against the risk of harm from false-positive findings or unwarranted treatment, particularly when incremental clinical benefit remains uncertain.

In summary, this survey highlights substantial variability in CNS imaging practices for aRCC, particularly among neurologically asymptomatic patients, underscoring the need for more standardised clinical pathways to guide decision-making. Optimised and standardised imaging protocols, including appropriate use of contrast-enhanced MRI and CT, may improve detection of occult brain metastases and enable earlier intervention when disease is more amenable to SRS and contemporary systemic therapies, with potential benefits for neurological outcomes, quality of life, and survival [5,11-13,16-18]. Emerging multi-institutional data reporting a 4.3% incidence of occult brain metastases suggest that routine CNS screening may warrant consideration even among patients with favourable IMDC risk profiles [6,11].

Consistent with our findings, recent literature increasingly calls for reassessment of CNS imaging strategies in aRCC. Recent multi-institutional series reports a clinically meaningful prevalence of occult brain metastases, challenging the longstanding assumption that CNS involvement is rare in asymptomatic patients and supporting more systematic baseline brain imaging in selected populations [6,13,20]. Reviews of RCC brain metastases further argue that historical under-recognition of CNS disease is no longer justified in the era of effective local and systemic therapies and advocate improved detection through optimised neuroimaging strategies [18]. While guideline-oriented discussions increasingly acknowledge the potential underestimation of CNS disease burden and recommend brain imaging in patients with advanced or higher-risk disease [9], the optimal scope, frequency, and clinical impact of routine CNS imaging remain unresolved and represent important areas for future research.

This study has several strengths: it addresses a clinically relevant and timely question, has achieved a good response rate for a specialist clinician survey, presents results clearly, integrates findings with contemporary literature, and candidly reports practice variation and uncertainty. Several limitations must be emphasised. First, the sample size was small (n = 25), limiting precision and generalisability. Second, the cohort was restricted to UK centres and may not reflect international practice. Third, data were self-reported and therefore susceptible to recall and social-desirability bias. Fourth, although the instrument was piloted to optimise clarity and feasibility, it did not undergo formal psychometric validation (e.g., content validity assessment, construct validation or reliability testing), a limitation inherent to exploratory questionnaire studies that may permit differential interpretation of items. Finally, the survey did not capture patient‑level or outcome data, precluding assessment of the clinical impact of differing imaging strategies. Imaging decisions may be influenced by tumour grade, size, burden or genomic features not captured in this survey; consequently, the patterns described here should be regarded as descriptive of clinician practice rather than definitive guidance applicable to all patient subgroups.

Accordingly, our findings should be regarded as hypothesis-generating and descriptive of clinician practice rather than as evidence to support specific changes in policy or guideline recommendations. We therefore emphasise the need for prospective, patient‑level studies, ideally with validated instruments, clearly defined imaging protocols, and measured clinical and health‑economic outcomes, to determine the clinical effectiveness, cost‑effectiveness, and optimal scope and frequency of CNS imaging in aRCC.

## Conclusions

This survey demonstrates substantial variability in the approach to brain imaging for patients with aRCC among UK oncology consultants, highlighting a lack of consensus and the influence of individual clinical judgement, institutional practices, and resource availability. These findings underscore the need for prospective, high‑quality studies to clarify the clinical value, optimal timing, and appropriate modalities for brain imaging in this population. Robust evidence will be essential to inform the development of clear, evidence‑based guidelines, reduce practice variation, and support consistent, patient‑centred care. Standardised imaging strategies may improve early detection of brain metastases, optimise treatment selection, and enhance neurological outcomes, survival, and quality of life. Furthermore, identifying and addressing barriers to guideline implementation, such as resource constraints and concerns regarding overdiagnosis, will be crucial to the successful translation of research into routine practice.

Future prospective or registry‑based studies should incorporate tumour‑level variables (histological subtype and grade, primary tumour size, and intracranial/extracranial disease burden), documented institutional staging algorithms, and relevant genomic or syndromic predisposition data. Inclusion of these variables would enable risk‑stratified analyses of CNS imaging yield, robust assessment of clinical and patient‑reported outcomes, and formal health‑economic evaluation to inform stage‑ and biology‑specific imaging recommendations. Overall, this study provides a foundation for future research and practice development in the management of aRCC and highlights a clear need to re-evaluate existing guidelines and standardise the approach to CNS imaging.

## Appendices

Appendix A

The survey questionnaire regarding CNS imaging for RCC is detailed below.

1. Demographics

A. Which option best represents your current role? (Select one)

·      Consultant

·      Registrar (SpR)

·      Clinical fellow/staff grade

·      CNS

·      Other

B. For how many years have you been working within your current role? (Select one)

·      <5 years

·      5-10 years

·      11-15 years

·      16-20 years

·      21-25 years

·      >25 years

C. Please indicate the name of the trust, hospital, or medical institution where you currently work (free-text answer)

D. How many consultants treat RCC in your local centre? (Select one)

·      1

·      2

·      3

·      4

·      5

·      >5

E. On average, in your practice, how many new RCC patients do you have per month? (Select one)

·      <4

·      4-6

·      7-9

·      >9

2. What Guidelines Are You Using for CNS Imaging in Metastatic RCC?

A. Which clinical guideline do you primarily follow for CNS imaging in investigating metastatic RCC? (Select one)

·      NCCN (National Comprehensive Cancer Network)

·      EAU (European Association of Urology)

·      ASCO (American Society of Clinical Oncology)

·      Institutional/Local Guidelines

·      No specific guidelines

·      Other (free-text response):

B. According to the guidelines you follow, when is CNS imaging recommended? (Select one)

·      Only when neurological symptoms are present

·      Routine baseline imaging at diagnosis of metastatic RCC

·      Periodic imaging regardless of symptoms

·      Imaging based on specific high-risk features (e.g., sarcomatoid differentiation, lung metastases, etc.)

·      At the point of disease progression

·       Other (free-text response):

3. What Are You Currently Doing in Your Practice?

A. How often do you perform CNS imaging in patients with metastatic RCC without known brain metastases? (Select one)

·      Only when neurological symptoms are present

·      Routine baseline imaging at diagnosis of metastatic disease

·      Periodic surveillance imaging (e.g., every 6-12 months)

·      Imaging in patients with high-risk features, even if asymptomatic

·      At the point of disease progression/treatment failure

·      Other (free-text response):

B. What imaging modality do you primarily use in CNS evaluation for metastatic RCC? (Select one)

·      MRI of the brain with contrast

·      CT scan of the brain with contrast

·      Both MRI and CT, depending on the situation

·      Other (free-text response):

C. In your experience, how often do you detect asymptomatic brain metastases in your RCC patients? (Select one)

·      >75% of the time

·      50-74% of the time

·      25-49% of the time

·      10-24% of the time

·      <10% of the time

·      Not applicable (I currently do not perform any CNS imaging in asymptomatic patients)

4. Are You Willing to Change Your CNS Imaging Practices?

A. Would you consider adopting routine CNS imaging for all metastatic RCC patients if evidence suggested it improved outcomes? (Select One)

·      Yes

·      No

·      Maybe (depending on the strength of the evidence)

B. What factors would influence your decision to change your CNS imaging practices? (Select all that apply)

·      Strong clinical trial evidence supporting routine imaging

·      Updated national or international guideline recommendations

·      Improved access to imaging resources (e.g., MRI availability, insurance coverage)

·      Evidence showing the cost-effectiveness of routine imaging

·      Feedback from multidisciplinary teams (e.g., tumour boards)

·      Patient preference for more comprehensive imaging

·      Other (free-text response):

C. What reasons, if any, would you have for not doing routine, increased frequency brain imaging in those with previously identified brain metastases? (Select all that apply)

·      Overdiagnosis leading to unnecessary interventions

·      Increased patient anxiety

·      Cost and resource allocation

·      Lack of clear benefit in survival or quality of life

·      Logistical challenges (e.g., scheduling, MRI access)

·      No reason- I am happy with my current frequency of CNS imaging

·       Other (free-text response):

D. If you planned to do routine CNS imaging in asymptomatic patients, what statement would best match your opinion on the frequency of CNS imaging? (Select one)

·      I would not perform routine imaging

·      I would perform routine CNS imaging every 3 months.

·      I would perform routine CNS imaging 6 months

·      I would perform routine CNS imaging every 12 months

·      I would perform routine CNS imaging with each new treatment line

E. What reasons, if any, would you have for not doing routine, increased frequency brain imaging in asymptomatic patients without previously identified brain metastases? (Select all that apply)

·      Overdiagnosis leading to unnecessary interventions

·      Increased patient anxiety

·      Cost and resource allocation

·      Lack of clear benefit in survival or quality of life

·      Logistical challenges (e.g., scheduling, MRI access)

·      Concern re: issues surrounding driving rules/DVLA restrictions if brain metastases are identified

·      No reason- I am happy with my current frequency of CNS imaging

·      Other (free-text response):
